# Put to the test

**DOI:** 10.7554/eLife.24276

**Published:** 2017-01-30

**Authors:** Marites T Woon, Timothy J Kamp

**Affiliations:** 1 Department of Medicine, the Cellular and Molecular Arrhythmia Research Program and the Stem Cell & Regenerative Medicine Center, University of Wisconsin-Madison, Madison, United States; 2 Department of Medicine, the Cellular and Molecular Arrhythmia Research Program, the Stem Cell & Regenerative Medicine Center and the Department of Cell and Regenerative Biology, University of Wisconsin-Madison, Madison, United Statestjk@medicine.wisc.edu

**Keywords:** induced pluripotent stem cells, arrhythmia, cardiotoxicity, Human

## Abstract

Personalized heart muscle cells made from stem cells in the laboratory could be used to check an individual’s response to potential new drugs before clinical trials.

**Related research article** Stillitano F, Hansen J, Kong C-W, Karakikes I, Funck-Brentano C, Geng L, Scott S, Reynier S, Wu M, Valogne Y, Desseaux C, Salem J-E, Jeziorowksa D, Zhar N, Li R, Iyengar R, Hajjar RJ, Hulot J-S. 2017. Modeling suceptibility to drug-induced long QT with a panel of subject-specific induced pluripotent stem cells. *eLife* **6**:e19406. doi: 10.7554/eLife.19406

Checking that a new drug candidate does not trigger a potentially lethal heart arrhythmia called torsades de pointes has become an essential and costly part of the drug development process over the past two decades. A drug-induced increase in the length of time it takes for heart muscle cells to reset so that they are ready for the next heartbeat, which is reflected by an increase in the QT interval on the surface electrocardiogram (ECG), is associated with an increased risk of torsades de pointes.

Extensive research has revealed that the most common cause for drug-induced increases in the QT interval is the blocking of a potassium ion channel called hERG (Roden, 2016). As a result, early in the drug development process, candidate small molecules are routinely screened for their ability to block hERG channels expressed in stable cell lines. Although this strategy has dramatically reduced the number of potentially harmful compounds that make it to clinical trials, it is costly in terms of time and resources. Furthermore, it eliminates many promising therapeutic compounds that may not actually cause torsades de pointes (Gintant et al., 2016). Indeed, some hERG blocking drugs, such as verapamil, do not increase the QT interval (Zhang et al., 1999), and there are various drugs that can increase the QT interval without causing torsades de pointes (such as ranolazine; Tzeis and Andrikopoulos, 2012). There is, therefore, a need for more cost effective and predictive models to address this safety issue.

Now, in eLife, Jean-Sebastien Hulot at the Icahn School of Medicine at Mount Sinai and colleagues – including Francesca Stillitano and Jens Hansen as joint first authors – report that heart muscle cells made from induced pluripotent stem cells might offer an answer to this problem (Stillitano et al., 2017).

Reprogramming somatic cells to become master stem cells called induced pluripotent stem cells (iPSCs for short) now provides the opportunity to generate any specific cell type present in the body that is genetically matched to the starting somatic cell (Takahashi et al., 2007; Yu et al., 2007). Previous studies have shown that heart muscle cells made from iPSCs from patients with inherited forms of long QT syndrome behave similarly to their own heart muscle cells (Moretti et al., 2010; Itzhaki et al., 2011). This suggests that iPSC-derived cells could be used as a model for evaluating whether potential new drugs might alter the QT interval in humans (Anson et al., 2011).

However, like all model systems, there are limitations. Most importantly, the heart muscle cells made by iPSCs are more similar to the heart muscle cells found in embryos than those found in adults (Yang et al., 2014), which may mean that they respond differently to drugs. A growing number of studies suggest that heart muscle cells produced by iPSCs do respond appropriately to drugs that are known to increase the QT interval in humans (Kitaguchi et al., 2016): however, we do not know whether these cells will faithfully reflect the responses of individual patients.

Stillitano et al. – who are based at Icahn, Sorbonne Universités and Cellectis Stem Cells – compare the responses of individual volunteers to a hERG blocking drug with the responses of iPSC-derived heart muscle cells generated from the same individuals (Figure 1). To do this, the team recruited 92 healthy and genetically diverse volunteers with normal baseline QT intervals and gave them a single dose of a drug called sotalol.

**Figure 1.**
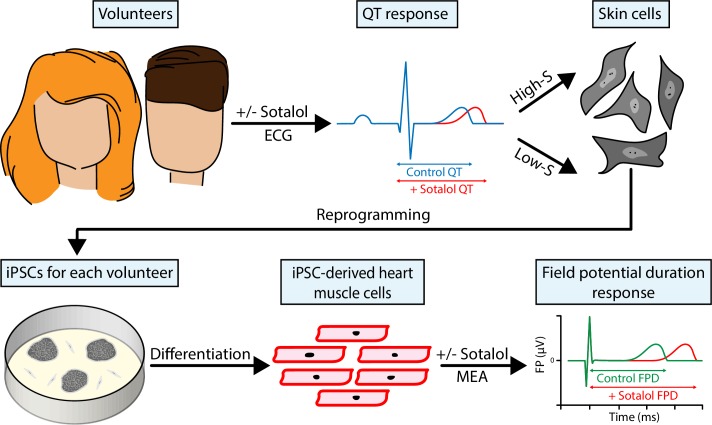
Reprogrammed skin cells can be used as models to test the cardiac safety of drug compounds. Stillitano et al. gave healthy volunteers (top left) a single dose of the drug sotalol, and then used an electrocardiogram (ECG) to measure how the drug affected their QT interval, which represents the length of time it takes for their heart muscle cells to reset after they contract for a heartbeat. Skin samples were collected from the 10 volunteers with the highest sensitivity to sotalol and the 10 volunteers with the lowest sensitivity, and skin cells from each of the volunteers in these two groups were reprogrammed to make induced pluripotent stem cells (iPSCs; bottom left). These iPSCs were then exposed to conditions that caused them to differentiate and become heart muscle cells. Finally, multielectrode arrays (MEA; bottom right) were used to measure changes in field potential duration (which is a surrogate for the QT interval) in the cells.

As anticipated, some individuals were more sensitive to sotalol than others. Stillitano et al. then focused on the 10 volunteers with the lowest sensitivity to the drug (that is, those with the smallest increases in QT intervals) and the 10 volunteers with the highest sensitivity. Skin cells from both groups were reprogrammed to make iPSCs, and these were then used to make heart muscle cells. The cells from the high sensitivity group had larger increases in a quantity called the field potential duration (which is a surrogate for the QT interval in cell studies) in response to sotalol than those from the low sensitivity group.

One might predict that the cells in the high sensitivity group would also be more sensitive to other hERG blocking drugs, but there does not appear to be any difference in the responses of the two groups to a hERG blocker called E4031. Is this apparent difference in the way that cells respond to sotalol and E4031 caused by noise in the model system? Or is it the result of these drugs having distinct effects on the other ion channels and regulatory proteins that regulate the QT interval? For example, Stillitano et al. show that there are differences in the expression of several genes that regulate ion channels between the high and low sensitivity groups.

Although this work suggests that heart muscle cells produced by iPSCs may be useful models for testing the ability of potential new drugs to alter the QT interval in humans, important questions remain. Stillitano et al. removed cell lines from two of the volunteers that did not respond to E4031 from the study, and it is not clear whether these lines would confound the results. Furthermore, the responses of the iPSC-derived cells from the two groups of volunteers did not segregate perfectly. Was this due to imperfect reprogramming among the samples from different individuals and could characterizing more than one clone per subject overcome this limitation? Could better preparations of heart muscle cells improve the results? Would three dimensional heart tissue constructs composed of the multiple cell lineages present in the heart be better predictors of the volunteers’ responses to sotalol and other drugs?

Further analysis would ideally compare responses across all 92 volunteers involved in the study rather than just the most and least sensitive groups, but the realities of time and cost make such a study even more difficult. Nevertheless, this is the first clinical study to test the usefulness of iPSC-derived heart muscle cells as models of the response of specific individuals to drugs. In the case of drug-induced changes in QT intervals, these reprogrammed cells are found to be promisingly predictive. Future studies will undoubtedly improve the methodologies, and this model system will likely also prove valuable in testing other forms of drug-induced cardiotoxicity. This study begins to validate the concept that it may actually be possible to test how individuals would respond to drugs in clinical trials without putting them at risk.
